# Folium Hibisci Mutabilis extract suppresses M1 macrophage polarization through mitochondrial function enhancement in murine acute gouty arthritis

**DOI:** 10.1186/s13020-025-01081-6

**Published:** 2025-02-28

**Authors:** Yichen Zhao, Jiahui Zhang, Wei Yan, Ping Jiang, Juncheng Li, Haojun He, Honghong Ma, Yuxin Zhang, Kai Yang, Min Jiang, Xiaobing Xi

**Affiliations:** 1 Department of Orthopaedics, Shanghai Key Laboratory for Prevention and Treatment of Bone and Joint Diseases, Shanghai Institute of Traumatology and Orthopaedics, Ruijin Hospital, Shanghai Jiao Tong University School of Medicine, Shanghai, 200025 China; 2 Department of Clinical Laboratory, Wuxi Branch of Ruijin Hospital Shanghai Jiao Tong University School of Medicine, Wuxi, 214111 China; 3 Wenzhou Medical University, Wenzhou, 325035 China

**Keywords:** *Folium Hibisci Mutabilis*, Acute gouty arthritis, M1 macrophage, Mitochondrion, UQCRC1

## Abstract

**Background:**

Acute gout arthritis (AGA) is a common metabolic joint disease and urgently needs a safer alternative therapy due to the significant side effects from long-term use of primary medications. *Folium Hibisci Mutabilis*, a traditional medicinal herb, has demonstrated promising therapeutic efficacy in the clinical management of AGA, but its pharmacological mechanisms remain to be elucidated.

**Methods:**

*Folium Hibisci Mutabili* was isolated and refined *into* the *Folium Hibisci Mutabilis* Extract (FHME). Then, monosodium urate-induced AGA animal models were applied to identify the anti-inflammatory and analgesic effects of FHME in vivo through various techniques, including ultrasonography, Paw withdrawal thresholds, histological staining, etc. We used RNA-seq, qRT-PCR, ELISA, and flow cytometry to evaluate the efficacy of FHME on M1 polarization. Utilizing transmission electron microscope and oxygen consumption rate examinations in conjunction with Mito-Tracker staining, we observed the effects of FHME on mitochondrial morphology and function. Finally, we employed proteomics analysis, siRNA, qRT-PCR, western blot and other techniques to investigate the underlying mechanism of FHME's actions between the two phenotypes and the key targets.

**Results:**

We observed a notable reduction in inflammation and pain, as well as the decreased infiltration of inflammatory cells and expression of IL-1β in synovial tissue of AGA mice upon treatment with FHME. FHME suppressed TNF-α, IL-1β, iNOS, and IL-18 expression in BMDM-derived macrophages and inhibited the formation of F4/80^+^CD86^+^ cells. Mechanically, FHME protected mitochondrial morphology and stimulated the expression of key oxidative phosphorylation proteins, such as Ubiquinol Cytochrome c Reductase Core Protein I (UQCRC1), UQCRC2, CYCS, and NDUFA4. Additionally, it enhanced the activity of respiratory complex III, recovered cellular aerobic respiration under LPS and MSU induction. FHME lost its effect to downregulate M1 macrophage polarization with the presence of rotenone or si-UQCRC1. Finally, 10 compounds were identified from FHME having potential binding affinity with the UQCRC1 protein.

**Conclusions:**

The therapeutic potential of FHME for AGA is associated with the maintenance of mitochondrial function to inhibit M1 macrophage polarization, which is intimately linked to the UQCRC1. Our findings highlight the potential of *Folium Hibisci Mutabilis* as a safe and effective approach for AGA.

**Supplementary Information:**

The online version contains supplementary material available at 10.1186/s13020-025-01081-6.

## Background

Acute Gouty Arthritis (AGA) is regarded as aseptic arthritis. The sustained increase of uric acid in serum leads to the deposition of monosodium urate (MSU) crystals within joints, synovial membranes and other soft tissues subsequently causing acute inflammation in the pathogenesis of AGA [1]. Based on the survey data in 2020, an estimated 55.8 million individuals worldwide were influenced by gout, and a forecasting model suggests the number could potentially escalate to nearly 95.8 million by 2050 [2]. The guidelines released by American College of Rheumatology and European League against Rheumatism recommend nonsteroidal anti-inflammatory drugs, colchicine, and glucocorticoids as first-line treatments for AGA [3, 4]. However, potential adverse effects of those drugs such as gastrointestinal, renal toxicity, and cardiovascular risks necessitate the exploration of alternative therapeutic modalities with a more favorable safety profile. [5, 6].

It is widely acknowledged macrophages play a crucial role in the whole course of AGA [7]. MSU crystals trigger the activation of resident M0 macrophages to exhibit polarization and differentiate into pro-inflammatory M1 macrophages. This early inflammatory response is marked by the secretion of cytokines such as nitrogen monoxide (NO), interleukin 1β (IL-1β), interleukin 18 (IL-18) and tumor necrosis factor-α (TNF-α) within joints. Subsequently, a rapid influx of innate immune cells occurs, instigating a cascade of inflammatory responses [8, 9]. Recent research indicates that mitochondria function as a primary intracellular signaling platform involved in inflammatory processes throughout the progression of AGA [10–12]. The presence of MSU crystals significantly impacts mitochondrial bioenergetics, affecting basal respiration, maximal respiration, protein leakage, ATP production, and spare respiratory capacity in cells. This dysfunction leads to increased production of ROS, resulting in oxidative stress in macrophages [13]. Two studies have demonstrated that lipopolysaccharide (LPS) induction impairs the morphology and function of macrophage mitochondria, with reduced oxidative phosphorylation capacity driving macrophages towards M1 polarization [14, 15]. Another study has highlighted that regulating cellular energy metabolism through mitochondria is crucial for IL-1β secretion [16]. Additionally, the scientific literature increasingly suggests that addressing mitochondrial damage may alleviate symptoms associated with AGA [17–19].

*Folium Hibisci Mutabilis* is the dried leaf of *Hibiscus mutabilis L.* and has been known as a traditional Chinese herb possessing the heat-clearing effect for thousands of years in China [20]. It is the major herbal component of detumescence powder, one of the therapeutic drugs of AGA in Ruijin Hospital. Unfortunately, little research investigating the pharmacological mechanisms underlying its therapeutic effects on AGA. A few results prove the extract of *Folium Hibisci Mutabilis* (FHME) inhibits the release of M1 macrophages’ markers such as TNF-α and NO in vitro [21]. In this study, we used a comprehensive investigation into the pharmacological effects of FHME on AGA in vivo and explored the influence of FEME on macrophage polarization, mitochondrial function and the specific mechanisms involved. This implies restoring mitochondrial function may be another therapeutic target in AGA treatment.

## Methods

### Animals

Thirty male C57BL/6 J mice (24 ± 2 g) at 8 weeks of age obtained from Zhejiang Vital River Laboratory Animal Technology Co., LTD was utilized to randomly assigned to either negative control group (treated with saline, Control, n = 6), AGA model group (treated with saline, Model, n = 6), AGA model with low concentration of FHME treatment group (treated with 400 mg/kg FHME, FHME.L, n = 6), AGA model with high concentration of FHME treatment group (treated with 800 mg/kg FHME, FHME.H, n = 6) or AGA model with colchicine treatment group (treated with 1 mg/kg colchicine, Col, n = 6), as positive control. The AGA model mice were established by injection of 3% MSU suspension (0.6 mg/20 μl) in the right hind paw, while negative control group received 20 μl saline solution injection at the same site. Subsequently, FHME and colchicine were given intragastrically for seven days. All mice in the study were housed in an environment with a 12/12-h light/dark cycle, a humidity of 55%−60%, and a temperature of 22–24 °C.

### Preparation of FHME and chemical component profiling

The FHME in the experiment was purchased from Shanghai Kangqiao Traditional Chinese Medicine Herbal Pieces Co., Ltd. The dried leaves were ground into powder and then extracted twice with 70% ethanol, each extraction lasting for 1 h. Following the combination of the two extracts, they were concentrated and then subjected to drying. Based on the dosage of human drugs used in clinical practice, the dose of FHME was converted to an equivalent dose for mice, with the conversion coefficient derived from the body surface area ratio between humans and mice.

To preliminarily identify the chemical composition of FHME, the powder of *Folium Hibisci Mutabilis* leaf was dissolved in purified water at a concentration of 10 mg/mL, followed by 1 h of ultrasonication and centrifugation at 14,000 rpm for 10 min. The collected supernatant was then resuspended in 0.1% formic acid and acetonitrile. We set operating parameters at 35 °C, 0.35 mL/min and obtained data in negative ion mode, with a scanning range from *m/z* 100 to 1700. Subsequent secondary mass spectrometry analyses were derived based on the data-dependent acquisition from the precursor ion list.

### Reagents and antibodies

Minimum essential medium alpha (α-MEM, #12,561,056), fetal bovine serum (FBS, #10,099,158), penicillin and streptomycin (PS, #15,140,122), TRIZOL reagent (#15596018CN), Lipo3000 (#L3000001) were both purchased from ThermoFisher (Waltham, USA). Macrophage Colony-Stimulating Factor (M-CSF, #CB34) was obtained from Novoprotein Scientific Inc (Suzhou, China). Lipopolysaccharides (LPS, #L2630), Monosodium Urate (MSU, #U2875), DAPI (#D9542) were purchased from Sigma (St. Louis, USA). Dimethyl Sulfoxide (DMSO, #632,408), Cell Counting Kit-8 kit (CCK8, #CK04) were purchased from Dojindo (Kyushu Island, Japan). IL-1β ELISA Kit (#PI301), TNF-α ELISA Kit (#PT512) and Mito-Tracker Deep Red FM (#C1032) were purchased from Beyotime (Suzhou, China). Antibodies for FITC anti-mouse F4/80 (#123,108), FITC Rat IgG1 (#400,406), APC anti-mouse CD86 (#105,012), APC Rat IgG1 (#400,411), Purified anti-mouse CD16/32 Antibody (#101,302), anti-CD206 (#141,707) and anti-CD11b (#101,203) were purchased from BioLegend (San Diego, USA). Antibodies for anti-iNOS (#ab283655), anti-IL-1β (#ab200478) and anti-beta actin (#ab8227) were commercially provided by Abcam (Cambridge, UK). Anti-F4/80 antibody (#21,705–1-AP) was purchased from Proteintech (Chicago, USA).

### Ultrasonography and paw thickness measurement

Ultrasonographic analysis of mice's hind paws was conducted using B-mode ultrasound imaging after seven days post-treatment with their hind paws were positioned on the ultrasound table and adjusted to ensure fully exposed and maintained in an optimal position parallel to the ultrasound probe. Daily measurements of the hind paw thickness were taken by the vernier caliper, which was recorded in millimeters (mm).

### Bone micro‐CT

Following a seven-day period of post-treatment, the right hind paws were immersed in 4% paraformaldehyde for 3 days and then preserved in 75% ethanol. Thereafter, an examination was conducted using the Micro-CT system (Skyscan 1A172, Belgium).

### Paw withdrawal thresholds (PWT)

Paw withdrawal thresholds in response to mechanical stimuli were determined utilizing von Frey filaments (Stoelting, USA) to quantitatively assess nociceptive sensitivity in mice. The evaluation commenced with the application of the 0.02 g filament to the plantar surface of the hind paw, incrementally increasing until a withdrawal threshold was attained. A positive response was characterized by the observance of withdrawal, shaking, or licking of the hind paw subsequent to filament application. The mechanical withdrawal threshold was calculated through the method delineated by Chaplan et al. [22].

### Hematoxylin–eosin (HE) staining

Upon euthanasia of the mice, tissues including the heart, liver, spleen, lungs, kidneys, and hind paws were carefully excised and immediately immersed in 4% paraformaldehyde for fixation. The hind paws were additionally decalcified using EDTA decalcification solution. The fixed specimens were then embedded in paraffin and sectioned. The paraffin sections were then sequentially deparaffinized by placing them in a gradient concentration of xylene and ethanol solution, followed by a wash with water to remove any residual reagents. The sections were subsequently stained with hematoxylin and eosin in a sequential manner before being permanently sealed. Microscopic observation analysis and image capture were conducted to assess the histological characteristics of the samples.

### Assessment of hepatic and renal function

Blood was carefully obtained from the orbital vessels of mice, allowing it to clot for 20 min. Following coagulation, serum was separated by centrifuging at 4 °C and 3000 rpm for 15 min. The levels of aspartate aminotransferase (AST), alanine aminotransferase (ALT), blood urea nitrogen (BUN), and creatinine (CREA) in the serum of each mouse group were then measured according to the instructions provided with the assay kits.

### Immunohistochemical (IHC) staining and immunofluorescence (IF) staining

The paraffin-embedded hind paw sections underwent dewaxing, antigen retrieval, and blocking with serum. Immunohistochemical staining was performed using antibodies specific to IL-1β and UQCRC1.

For immunofluorescence staining, the primary antibodies CD86 and F4/80 were diluted according to the manufacturer's instructions, incubated overnight at 4 °C, and subsequently washed three times with PBS. The samples were then incubated with the corresponding fluorescent secondary antibodies at room temperature, shielded from light, for a duration of 1 h, followed by another three washes with PBS. Finally, cellular nuclei were counterstained with DAPI. Image acquisition and analysis were carried out using a fluorescence microscope.

### Cell culture

The femurs and tibias from six-week-old male C57BL/6 J mice were meticulously extracted and underwent a series of rigorous cleansing and rinsing steps to isolate bone marrow-derived monocytes (BMDMs). These cells were then incubated in α-MEM medium with 10% FBS, 100 U/ml penicillin, 100 μg/ml streptomycin, and maintained at 37 °C in an atmosphere of 95% humidity with 5% CO₂ for a duration of 24 h. Following this initial incubation period, 20 ng/mL M-CSF was added to the supernatant cells. After an additional 48-h incubation, the cells that had adhered to the bottom of the petri dish were classified as BMDMs. Subsequently, these adherent cells were harvested and seeded onto porous culture dishes. We used 500 ng/ml LPS and 300 μg/ml MSU to stimulate to differentiate into polarized macrophages under the specific experiments’ requirements.

### CCK8 assay

BMDMs were seeded in 96-well plates at a density of 3 × 10^3^ cells/well and allowed to adhere overnight. Then, the cells were exposed to FHME at concentrations of 0, 1, 5, 10, 50, 100, 200, 400, and 800 μg/ml in triplicates for 48 h to assess proliferation and survival. After treatment, CCK8 solution (10 μl/well) was added to each well and incubated at 37 ºC for 2 h. The optical density (OD) 450 nm was measured with a microplate reader (Tecan, Zurich) and cell viability was calculated according to OD value.

### Quantitative real-time PCR (qRT-PCR)

We utilized Trizol reagent and a cryo-centrifuge to extract total RNA from the cells. Following the manufacturer's guidelines (#RR036A, Takara), 1ug of total RNA was added to 20ul of reverse transcription system to generate cDNA. Subsequently, qRT-PCR was conducted using a kit (#RR420A, Takara) on an ABI 7500 Sequencing Detection System. The mRNA expression levels were normalized against β-actin mRNA. The primer sequences used in this study are listed in Table 1.

**Table 1:** Primer sequences for qRT-PCR

Primer	Primer sequence
TNF-α	Forward 5′-AGTGACAAGCCTGTAGCCC-3′
	Reverse 5′-GAGGTTGACTTTCTCCTGGTAT-3′
IL-1β	Forward 5′-CTGTGACTCATGGGATGATGATG-3′
	Reverse 5′-CGGAGCCTGTAGTGCAGTTG-3′
iNOS	Forward5′- GTTCTCAGCCCAACAATACAAGA-3′
	Reverse 5′- GTGGACGGGTCGATGTCAC-3′
IL-6	Forward5′- TAGTCCTTCCTACCCCAATTTCC-3′
	Reverse 5′- TTGGTCCTTAGCCACTCCTTC-3′
Arg1	Forward 5′- CTCCAAGCCAAAGTCCTTAGAG-3′
	Reverse 5′- AGGAGCTGTCATTAGGGACATC-3′
CD206	Forward 5′- CTCTGTTCAGCTATTGGACGC-3′
	Reverse 5′-CGGAATTTCTGGGATTCAGCTTC-3′
IL-10	Forward5′- GCTCTTACTGACTGGCATGAG-3′
	Reverse 5′- CGCAGCTCTAGGAGCATGTG-3′
Uqcrc1	Forward 5′- AGACCCAGGTCAGCATCTTG-3′
	Reverse 5′- GCCGATTCTTTGTTCCCTTGA-3′
Uqcrc2	Forward 5′- AAAGTTGCCCCGAAGGTTAAA-3′
	Reverse 5′-GAGCATAGTTTTCCAGAGAAGCA-3′
Cycs	Forward5′- CCAAATCTCCACGGTCTGTTC-3′
	Reverse 5′- ATCAGGGTATCCTCTCCCCAG-3′
Ndufa4	Forward5′- TCCCAGCTTGATTCCTCTCTT-3′
	Reverse 5′- GGGTTGTTCTTTCTGTCCCAG-3′
β-actin	Forward 5′-GGCTGTATTCCCCTCCATCG-3′
	Reverse 5′-CCAGTTGGTAACAATGCCATGT-3′

### Enzyme-linked immunosorbent assay (ELISA)

Following a 24-h incubation of the cultured cell, the cultured medium was isolated and subjected to centrifugation at 500 g for 5 min. Sandwich immunoassays were then employed to measure the levels of cytokine production in the cultured medium, specifically IL-1β and TNF-α, according to the manufacturer's guidelines.

### Flow cytometry analysis

BMDMs were washed twice with PBS and collected after 400 g centrifugation. Antibodies against F4/80 and CD86 were utilized to identify M1 macrophages, whereas antibodies against CD11b and CD206 were employed to detect M2 macrophages. In total, 2 × 10^5^ cells were incubated with the antibodies at room temperature for 20 min. Subsequently, cells were washed three times with PBS before determining the expression of the surface molecules using flow cytometry on BD Fortessa^TM^X-20. The data were analyzed using FlowJo Software (version 10.6). Initially, the Forward Scatter (FSC) and Side Scatter (SSC) parameters were employed to gate the cell populations. Adherent aggregates were then excluded based on the FSC-Height (FSC-H) and FSC-Area (FSC-A) parameters. False positives were identified and excluded by referencing the results from isotype control experiments. Following these steps, the proportion of positive cells was determined.

### RNA-seq analysis

For RNA-seq, BMDMs were exposed to LPS/MSU with 200 μg/ml FHME or an equivalent amount of DMSO as a control for 24 h. Following the extraction of total RNA, the concentration, purity, and integrity of the RNA were assessed. The cDNA library was then prepared, and the Illumina NovaSeq platform was used to sequence the transcriptome. Bioinformatics analysis was conducted using R (version 4.2.2). The differentially expressed genes (DEGs) between the model group and the FHME group were identified using the *limma* package. Genes with a *p*-value of less than 0.05 were selected as DEGs and were plotted on volcano maps. To evaluate alterations in immune cell infiltration, we utilized the CIBERSORT algorithm, which examined changes in the proportion of immune cell infiltration in AGA.

### Transmission electron microscope (TEM) examination

The cellular precipitate was harvested through centrifugation and subsequently immersed in an electron microscope fixative, where it was allowed to resuspend at 4 °C for a duration of 2–4 h. This process was followed by rinsing the precipitate with 0.1 mol/L phosphate buffer and encapsulating it within a heated 1% agarose solution. The encapsulated precipitate underwent further fixation in 1% osmium acid at 4 °C for an additional 2 h. The samples were then dehydrated using a gradient acetone solution placed in an embedding solution at 37 °C for 12 h, and cured in an oven at 37 °C overnight followed by another 48 h at 60 °C. Once cured, the samples were meticulously sectioned at thicknesses ranging between 60–180 nm. These sections underwent staining with 3% uranyl acetate and lead nitrate, ensuring careful handling and fixation at 4 °C. After staining, the samples were again cured at 60 °C for a duration of 48 h. In the final step, the stained samples were examined under a transmission electron microscope, from which images were collected and subjected to rigorous analysis.

### Mito-tracker staining

To prepare the working solution, the Mito-Tracker Deep Red FM solution was diluted in the cell culture medium at a ratio of 1:1000, achieving a final concentration of 400 nM. The cells were incubated with prepared Mito-Tracker Deep Red FM solution at 37 °C for 30 min to allow staining. Then the cells were fixed by 4% paraformaldehyde for 15 min, which was subsequently replaced with 0.2% Triton X-100 to permeabilize the cells for an additional 10 min. Following with the fluorescence quenching blocker that contained DAPI, observations and imaging of the cells were carried out using Zeiss 800 confocal microscope.

### Oxygen consumption rate(OCR) and extracellular acidification rate(ECAR)

Seahorse XF Cell Mito Stress Test Kit (#103,015–100, Agilent), Seahorse XF Glycolysis Stress Test Kit (#103,020–100, Agilent) and the Seahorse XF Analyzer (Agilent, USA) were employed to quantify OCR and ECAR. In the procedure, the OCR was determined by initially administering 1.5 μM oligomycin to ascertain the maximal respiratory capacity of cells for ATP production in the absence of phosphorylation constraints. Subsequently, a combination of 0.5 μM ROT/AA was introduced to gauge non-mitochondrial oxygen consumption. During ECAR measurement, a distinct sequence of reagents was sequentially introduced, including 25 mM glucose, 2 μM oligomycin, and 50 mM 2-DG (2-deoxyglucose).

### Protein gel strip mass spectrometry identification

Proteins from cell lysates were separated by SDS-PAGE according to molecular weight, and the excised gel bands underwent destaining, reduction, and alkylation before digestion with trypsin, which breaks the proteins down into peptides suitable for mass spectrometry analysis. These peptides were then identified using LC–MS/MS to provide detailed information on peptide mass and sequence. The MASCOT was employed to match the protein data against protein databases for identification. Further bioinformatic analyses of the mass spectrometry results elucidated the signaling pathways, biological processes, and interaction networks involved by the differentially expressed proteins.

### Western blotting

The cell lysate was separated by electrophoresis and transferred onto a PVDF membrane followed by incubating the membrane with the blocking solution at room temperature for 1 h. Subsequently, the membrane was incubated with primary antibody at 4 °C for 16 h, washed by TBST, and incubated with secondary antibodies at room temperature for 2 h. We visualized the protein bands using chemiluminescence reagents and ImageQuant LAS 5000 and finally analyzed the results employing Image J software.

### Mitochondrial complex III activity

The activity of Complex III, also named Coenzyme Q Cytochrome C Reductase, was measured using the Cell Mitochondrial Complex III Activity Assay Kit (#E-BC-K836-M, Elabscience). The protein concentration quantified by the BCA method was used to normalize enzyme activity, following the protocols and guidelines stipulated by the reagent manufacturer.

### Cell transfection

UQCRC1 siRNA was purchased from Shanghai Woze Biotechnology Co., Ltd. (Shanghai, China), including UQCRC1 siRNA1 (target sequence 5′-GCACAGACUUGACUGACUATT-3′), UQCRC1 siRNA2 (target sequence 5′-GCUAUGAGACUGAGAAGAATT-3′), UQCRC1 siRNA3 (target sequence 5′-GCAGAACAGUAGUCUGGAATT-3′). Lipo3000 was utilized to facilitate the transfection of UQCRC1 siRNA into BMDMs.

### Molecular docking

The structure of components of FHME was prepared by LigPrep tool from AutoDock Vina. The crystal structure of human respiratory supercomplex III bound with UQCRC1 was downloaded from RCSB PDB repository, namely PDB ID 5XTE [23]. Molecular docking was conducted using AutoDock Vina, and the corresponding binding energy data were calculated. The ten models exhibiting the lowest binding energies were visualized using PyMOL software.

### Statistical analysis

All data are presented as mean values with standard deviations (SD). Statistical significance is indicated as *P < 0.05, ** P < 0.01, *** for P < 0.001, utilizing a two-tailed non-paired Student’s t-test to assess differences.

## Results

### FHME provides relief from swelling and pain symptoms in AGA mice

**Fig. 1:**
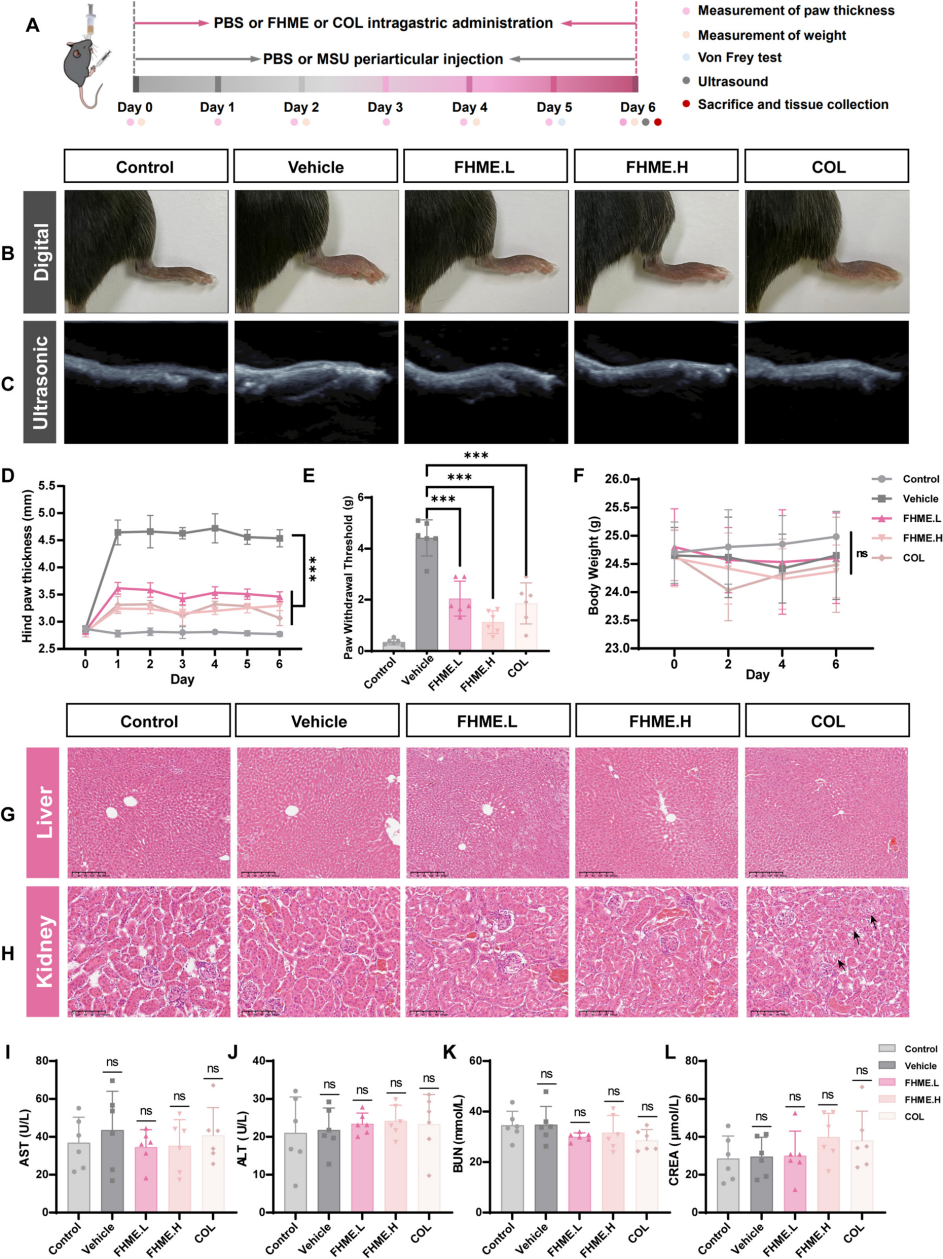
FHME provides relief from swelling and pain symptoms in AGA mice. **A** Schematic procedure of the FHME treated AGA mice model. Eight-week-old male C57BL/6 J mice were randomly assigned to five groups (n = 6 each group), either control group (treated with vehicle), vehicle group (treated with vehicle), FHME.L group (treated with 400 mg/kg FHME), FHME.H group (treated with 800 mg/kg FHME), or COL group (treated with 1 mg/kg colchicine). Control group was periarticular injected into the hind paw once a day for one week. Then 15 mg/kg MSU was injected to establish the gouty arthritis model following the same frequency. **B** Representative digital images of hind paws in mice. **C** The hind paw swelling in mice was assessed through ultrasonography, with the results presented in B-mode imaging. (**D**) The hind paw thicknesses were measured by vernier caliper daily. **E** The threshold for paw withdrawal in response to von Frey hair stimulation was assessed in each mouse. (**F**) The dynamic bodyweight changes. **G**, **H** Representative H&E-stained images of liver (**G**), and kidney (**H**). **I**–**L** The level of aspartate transaminase (AST, **I**), alanine aminotransferase (ALT, **J**), blood urea nitrogen (BUN, **K**), and Creatinine (CREA, **L**) in mice serum were identified to validate the toxicology of FHME. Data were calculated by two-tailed non-paired Student’s t-test, ***P < 0.001, ns, no significant differences

To construct AGA mice model, 20 µl suspension of 3% MSU was injected into the left paw of C57BL/6 J male mice, accompanied by intragastric administration of 400 mg/kg FHME (FHME.L) and 800 mg/kg (FHME.H) to determine the efficacy of FHME in AGA mice (Fig. 1A). The representative photographs and ultrasound images revealed that FHME alleviated the swelling of the hind paw compared with the vehicle group (Fig. 1B-C). These results were reconfirmed by statistical analysis of the thickness of the hind paw in the mice (Fig. 1E). As demonstrated by micro-CT, the joints in each group exhibited complete morphology, with no evidence of damage to the bone or cartilage (Fig.S1A). For evaluating alterations in pain perception after FHME administration, von Frey thresholds were applied and demonstrated a significant increase in thresholds in the FHME groups without weight loss (Fig. 1D, F). The safety of FHME was confirmed by histopathological examinations and serum analyses, revealing no significant pathological changes in the heart, liver, spleen and lung tissues compared to control group (Fig. 1G and S1B-C). Furthermore, no vacuolar degeneration was observed when treated with FHME, which differed from the COL group (indicated as black arrows) (Fig. 1H). The quantification of AST, ALT, BUN and CREA in the serum indicated that FHME did not affect liver and kidney function (Fig. 1I-L). These results suggested FHME could alleviate the symptoms of AGA mice without significant toxicity.

### *FHME inhibits the expression of inflammatory factors *in vivo* and *in vitro

**Fig. 2:**
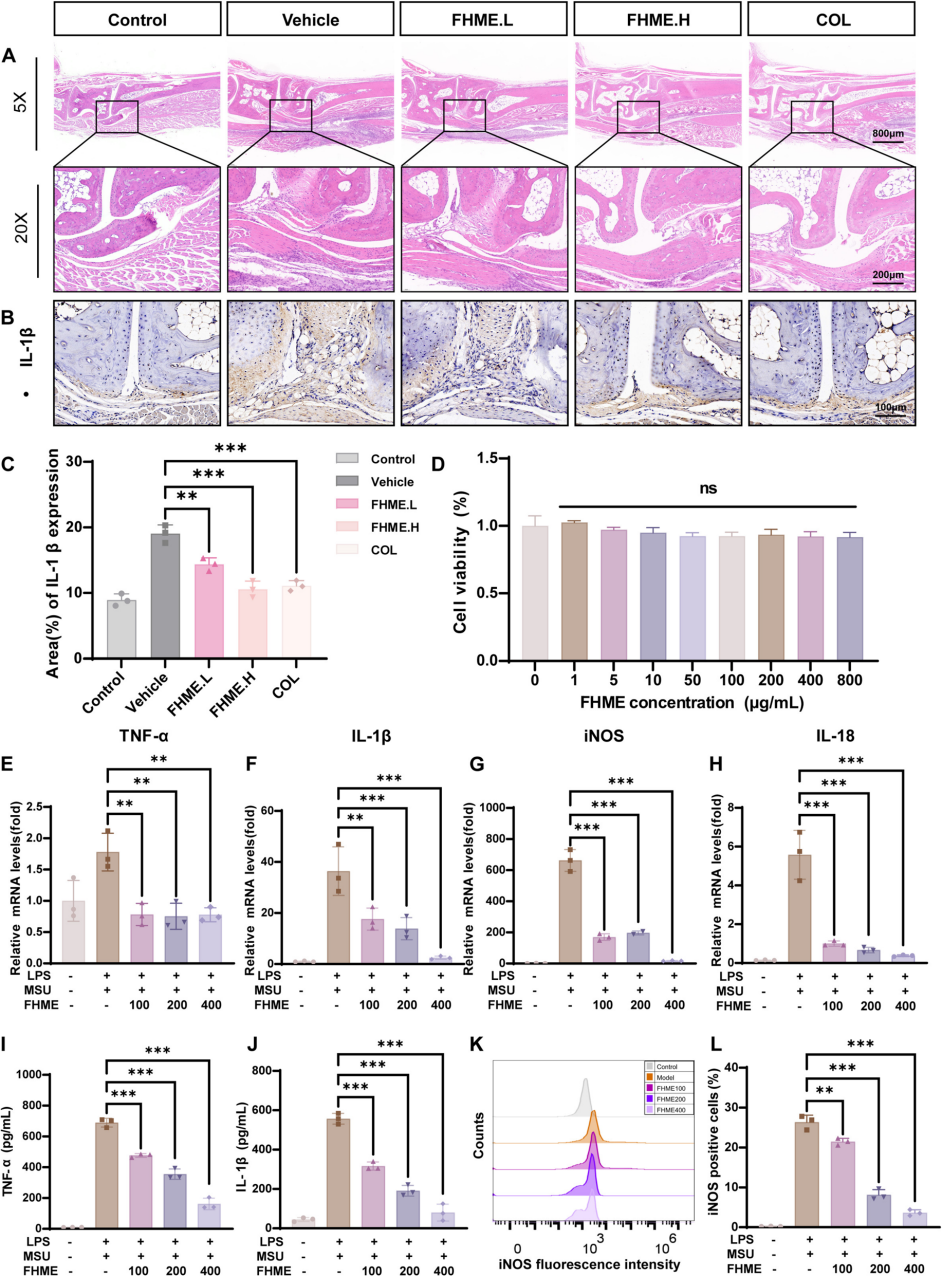
FHME inhibits the expression of inflammatory factors in vivo and in vitro. **A** Representative H&E-stained images of hind paw’s tissue sections. Original magnification encompassed 5 × and 20 × . **B** Representative images of immunohistochemical staining of IL-1β in the synovial tissues closing to hind paw metatarsophalangeal joint. **C** Statistics and analysis of the percentage of positive areas of IL-1β in B. **D** BMDMs maintained for proliferation by M-CSF were cultured with increasing doses of FHME for 48 h, then cell viability was determined by CCK8 assay. (**E** to **H**) M-CSF and LPS were incubated with BMDMs in the presence of increasing doses of FHME (100, 200 and 400 µg/mL) for 24 h, and gene levels of TNF-α (**E**), IL-1β (**F**), iNOS (**G**) andIL-18 (**H**) were determined by qRT-PCR analysis. (**I**, **J**) The concentrations of secreted TNF-α and (**I**) IL-1β (**J**) in the supernatant were quantified utilizing ELISA. (**K**) iNOS was detected using flow cytometry. **L** The percentage of iNOS-positive cells was statistically analyzed. Data were calculated by two-tailed non-paired Student’s t-test, **P < 0.01, and ***P < 0.001, ns, no significant differences

To further validate the mechanisms of FHME in AGA mice, histological examination was performed with ankle joint sections. The results revealed a significant inflammatory cell infiltration and synovial tissue disarray in vehicle group compared to Control group. However, FHME treatment reduced the number of infiltrating inflammatory cells in AGA mice (Fig. 2A). To further confirm the concentration of inflammatory factors in the joint, the expression of IL-1β was evaluated by immunohistochemistry. The vehicle group exhibited an accumulation of IL-1β around the ankle joint, which was effectively inhibited by FHME and colchicine administration (Fig. 2B, C). Therefore, we evaluated the anti-inflammation effect of FHME in vitro with BMDMs. The CCK8 assay indicated FHME did not induce any cytotoxic effects on BMDMs (Fig. 2D). We examined the mRNA expressions of inflammatory cytokines by qRT-PCR, including TNF-α, IL-1β, inducible nitric oxide synthase (iNOS), IL-18, and discovered that these genes were all upregulated in response to LPS/MSU stimulation at different stages, while FHME treatment significantly inhibited their expressions in a concentration-dependent manner (Fig. 2E–H). Additionally, we also utilized ELISA to quantify the concentrations of secreted TNF-α and IL-1β in the culture medium and found that the FHME inhibited the secretion of these factors under LPS/MSU stimulation (Fig. 2I, J). Flow cytometry analysis investigated a notable reduction in the number of iNOS^+^ cells (Fig. 2K, L). These findings suggest that FHME exhibits a promising anti-inflammatory effects both in vitro and in vivo.

### FHME attenuates M1 macrophage polarization

**Fig. 3:**
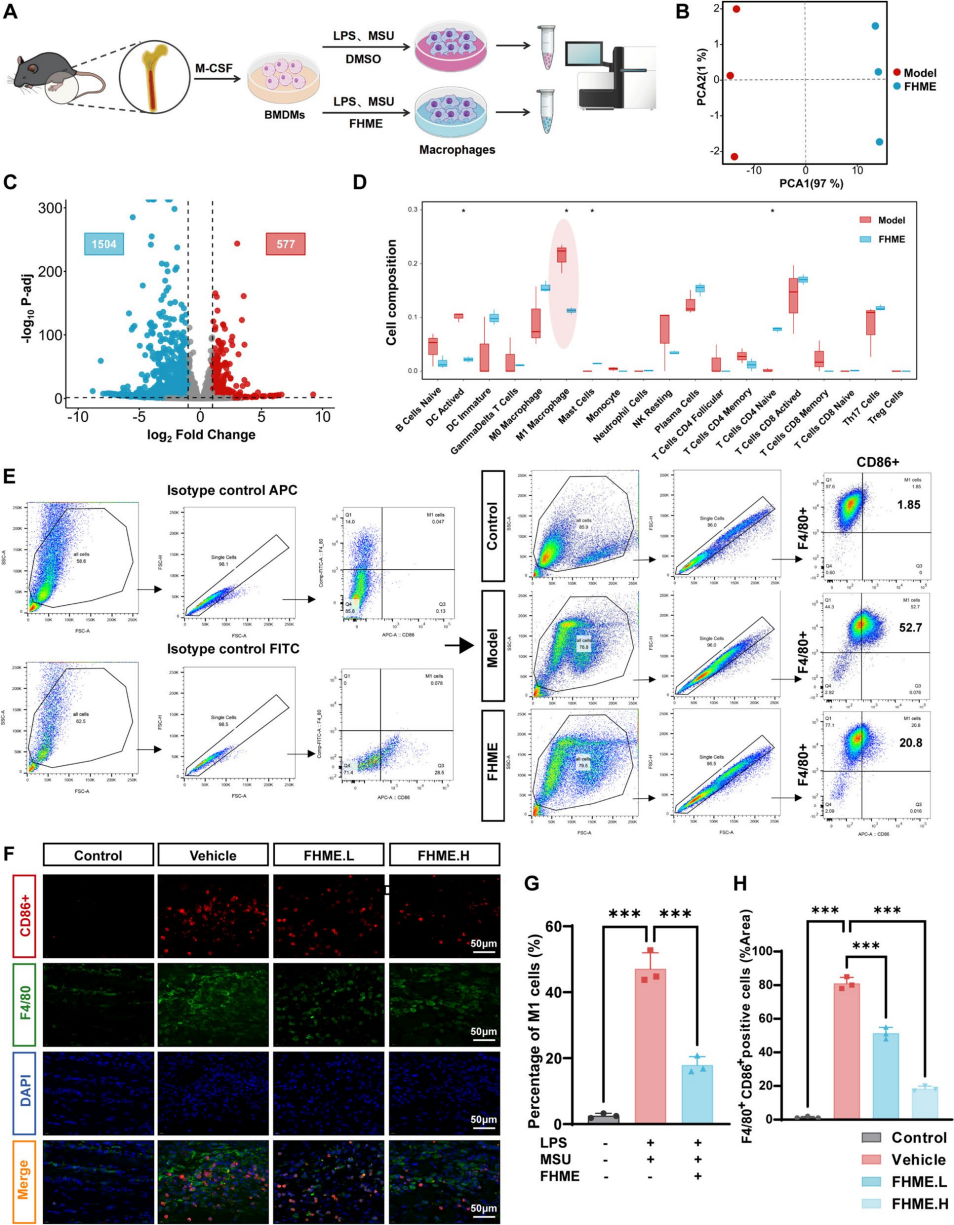
FHME attenuates M1 macrophage polarization. **A** Schematic workflow of RNA-seq for FHME treatment of LPS/MSU stimulated BMDMs. **B** Principal-component analysis reveals the transcriptomic divergence in BMDMs of Model group compared to FHME group. **C** The volcano plot displayed fold change (FC) and P-value of each gene calculated by DEGs analysis (group Model vs. group FHME). The red color highlights upregulated genes and blue color marks the downregulated genes. **D** Analysis of immune cell infiltration landscape, the histogram distinct in the scores of 19 immune cell types between model group and FHME group. **E** Isotypes controls for CD86-APC and F4/80-FITC were evaluated. F4/80 and CD86 were labeled by antibodies and detected by flow cytometry. **G** Statistics analysis of the percentage of F4/80^+^CD86^+^ cells in total cells. **F** Representative images of immunofluorescence staining indicated the location of F4/80^+^ and CD86^+^ cells in tissues. Merged images showed cells exhibiting dual fluorescence labeling, manifesting as orange under microscopic observation, **H** Analysis of the F4/80^+^CD86^+^ cells in each group of G. Data were calculated by Student’s t-test, *P < 0.05, and ***P < 0.001

The CIBERSORT algorithm functions in evaluating the immune infiltration and could be useful to identify which immunocytes were related to the therapeutic effect of FHME on AGA. Thus, LPS/MSU stimulated BMDMs treated with (FHME group, for 24 h) or without FHME (Model group) were subjected to RNA-seq analysis (Fig. 3A). Principal Component Analysis (PCA) revealed notable gene expression differences between Model and FHME groups (Fig. 3B). The volcanic diagram indicated 577 upregulated and 1504 downregulated genes (Fig. 3C). Then, we employed CIBERSORT algorithm to elucidate the correlation among 19 distinct immune cell infiltrations, and the results indicating DC activated and M1 macrophage showing a lower infiltration after FHME treatment compared with Model group. Conversely, mast cells and T cells CD4 naive exhibited elevated infiltration. Among these four types of immune cells, M1 macrophage constituted the highest composition (Fig. 3D).

Undifferentiated M0 macrophages can polarize to M1 or M2 in the pathogenesis of AGA [9]. Among of which, M1 macrophage polarization plays a pivotal role in being capable of secreting pro-inflammatory cytokines and mediators. To prove the impact of FHME on M1 macrophage polarization in vitro, BMDMs were treated with 200 µg/mL FHME for 24 h, followed by flow cytometry analysis. The results showed a decreased CD86^+^F4/80^+^ cells in FHME group, compared to that in Model group (Fig. 3E and G). Immunofluorescence staining was employed to examine the F4/80^+^CD86^+^ cells in hind paw tissues, and revealed a substantial decrease in FHME administrated AGA model mice (Fig. 3F and H). The balance of M1/M2 is important in the occurrence and recovery of inflammation. M2 macrophages have the function of reducing inflammation and achieving local homeostasis. Therefore, we also analyzed the effect of FHME on M2 polarization under IL-4 and IL-13 stimulation. The qRT-PCR results indicated that FHME did not exert a significant influence on the mRNA expression levels of Arg1, CD206 and IL-10, which are recognized as M2 macrophage markers (Fig.S2A-C). Furthermore, flow cytometry results also suggested FHME had no enhancement in CD11b^+^CD206^+^ cells (Fig.S2D-E).

### FHME restores mitochondrial stability and improves energy metabolism to reverse LPS/MSU induced M1 polarization

**Fig. 4:**
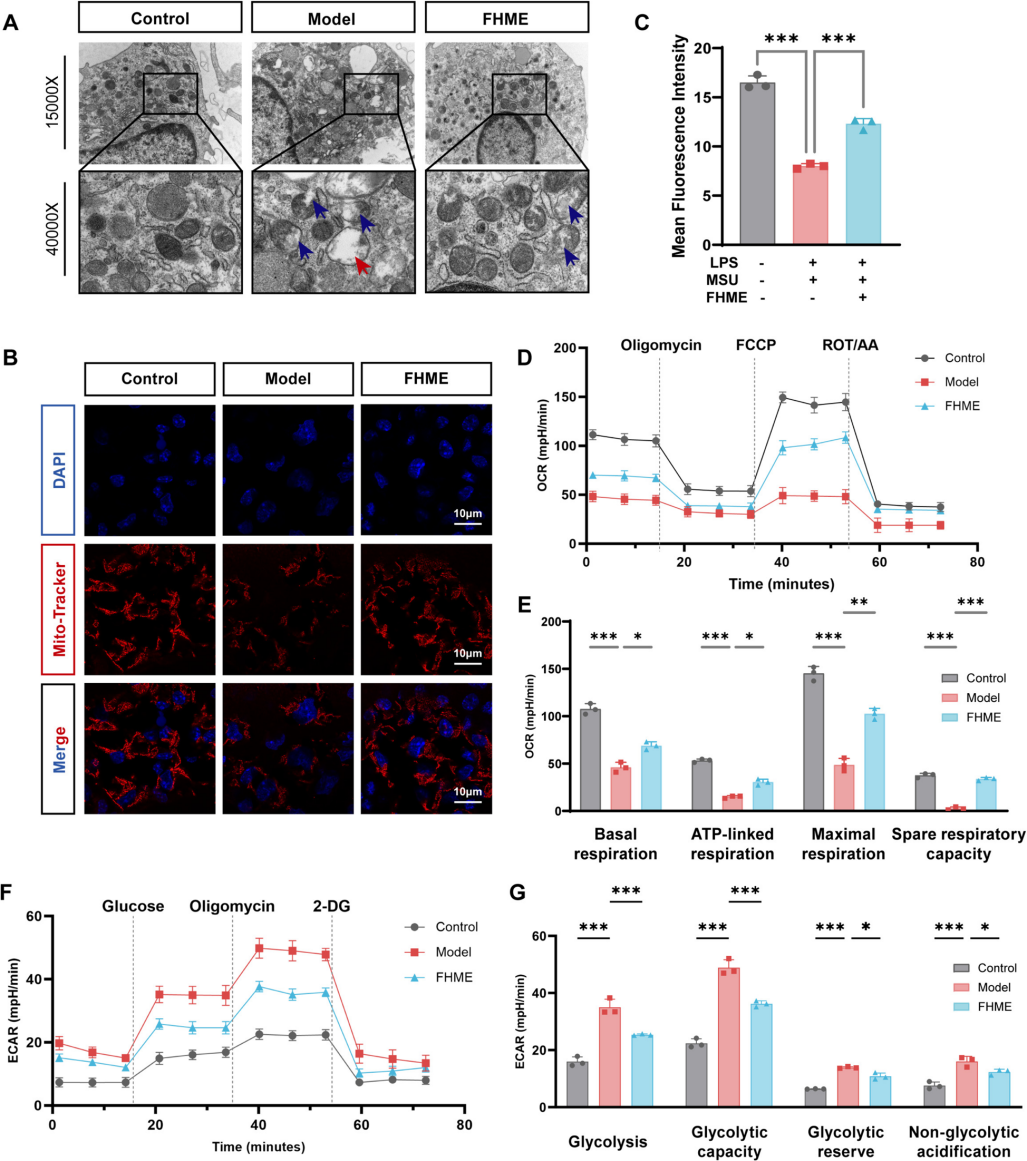
FHME restores mitochondrial stability and improves energy metabolism to reverse LPS/MSU induced M1 polarization. BMDMs were randomly assigned to three groups. Control group was added M-CSF. Model group and FHME group were incubated with M-CSF, LPS/MSU in the presence or absence of 200 µg/mL FHME for 24 h. **A** Representative transmission electron microscopy images of mitochondrial morphology (× 15,000, × 40,000) in cells. The arrow symbol represented abnormal mitochondria, characterized by bubble change (red arrow), broken ridge and membrane rupture (blue arrow). **B** Representative Mito-Tracker Red fluorescence images observed the phenotype and morphological changes of mitochondria. **C** Quantitative analysis of fluorescence intensity of the cells in B. **D** Alterations in the mitochondrial oxygen consumption rate (OCR) were observed following treatment with FHME. **E** Statistics analysis of the basal respiration, ATP-linked respiration, maximal respiration, and spare respiration capacity in OCR. **F** Extracellular acidification rate (ECAR) was measured to quantify glycolytic capacity. **G** Statistics analysis of the glycolysis, glycolytic capacity, glycolytic reserve, and non-glycolytic acidification in ECAR. Data were calculated by Student’s t-test, *P < 0.05, **P < 0.01, and ***P < 0.001

Mitochondrial damage and abnormal energy metabolism have been reported to be important factors influencing macrophage polarization [24, 25]. Therefore, to further explore the mechanism of M1 polarization reversal by FHME, we investigated the protective effect of FHME against mitochondrial damage and abnormal energy metabolism during M1 polarization in vitro. Firstly, we employed electron microscopy to validate the protective effects of FHME on mitochondria structure during M1 polarization. The results revealed that culturing BMDMs with LPS/MSU led to extensive mitochondrial damage, characterized by increased mitochondrial size, a disorganized arrangement and the reduced mitochondrial density (Fig. 4A). However, FHME treatment reorganized the mitochondrial arrangement and reduced the formation of bubble change, broken ridge and membrane rupture (Fig. 4A). Further results in Mito-tracker red staining also displayed FHME rescued the reduction of mitochondrial membrane potential induced by LPS/MSU stimulation (Fig. 4B, C).

Additionally, we also detected the OCR and ECAR functions of mitochondria in these cells. LPS/MSU stimulation resulted in a decrease in basal respiration, an effect primarily due to diminished ATP-linked respiration, maximal respiration, and spare respiratory capacity. These alterations are indicative of a reduction in the maximum activity of the electron transport chain. Notably, FHME treatment reversed these changes (Fig. 4D, E). Under inflammatory conditions, glycolysis, an alternate energy metabolism pathway, serves as a principal pathway through which macrophages generate a substantial quantity of ATP. To further elucidate the impact of FHME on glycolysis, we examined its influence on various glycolytic parameters, including glycolysis, glycolytic capacity, glycolytic reserve, and non-glycolytic acidification, when stimulated by LPS/MSU. Quantitative analyses revealed that FHME markedly decreased all four phases of ECAR (Fig. 4F, G). These findings imply that FHME suppresses anaerobic respiration and can effectively preserve mitochondrial structure and functions.

### FHME relies on mitochondrial function to inhibit M1 macrophage polarization

**Fig. 5:**
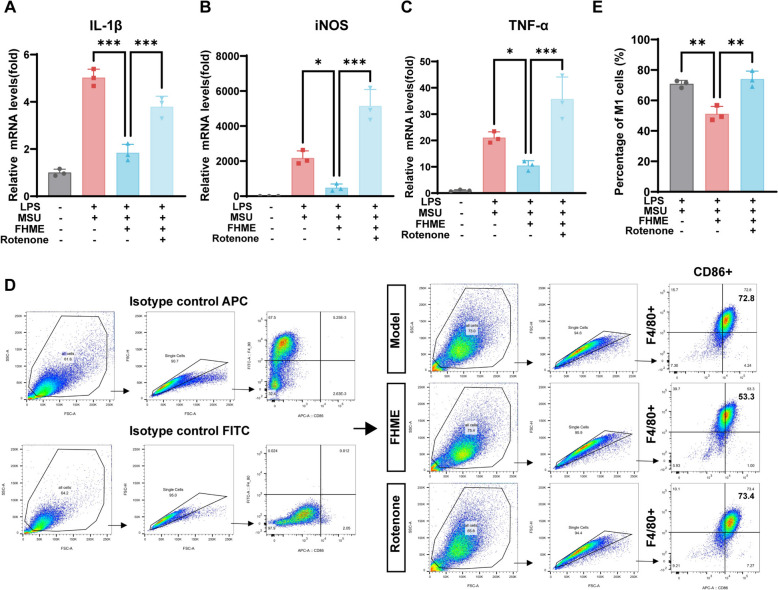
FHME relies on mitochondrial function to inhibit M1 macrophage polarization. **A**–**C** BMDMs were incubated with LPS, MSU and FHME in the presence or absence of 0.5 μM rotenone for 24 h. Gene levels of IL-1β (A), iNOS (**B**) and TNF-α (**C**) were determined by qRT-PCR analysis. **D** Isotypes controls for CD86-APC and F4/80-FITC were evaluated. F4/80 and CD86 were labeled by antibodies and detected by flow cytometry. **E** The percentages of F4/80^+^CD86^+^ cells among the total cell count were statistically assessed. Data were calculated by Student’s t-test, *P < 0.05, **P < 0.01, and ***P < 0.001

To investigate whether FHME inhibits M1 macrophage polarization through the mitochondrial homeostasis, we employed rotenone, which inhibits electron transfer from NADH to CoQ, thereby impairing mitochondrial function [26]. Subsequently, we co-incubated rotenone with FHME under stimulation by LPS/MSU. The results indicated that rotenone could totally withdraw the protecting capability of FHME from LPS/MSU stimulation, revealed by increased expression of inflammation factors, including IL-1β, iNOS and TNF-α (Fig. 5A–C). Furthermore, flow cytometry experiments also revealed a increased F4/80^+^CD86^+^ M1 macrophage cells after rotenone treatment (Fig. 5D, E). Thus, these results indicated that FHME indeed targets in protecting the mitochondrial function to reverse the LPS/MSU induced M1 polarization in AGA model.

### FHME promotes UQCRC1 expression to sustain mitochondrial function

**Fig. 6:**
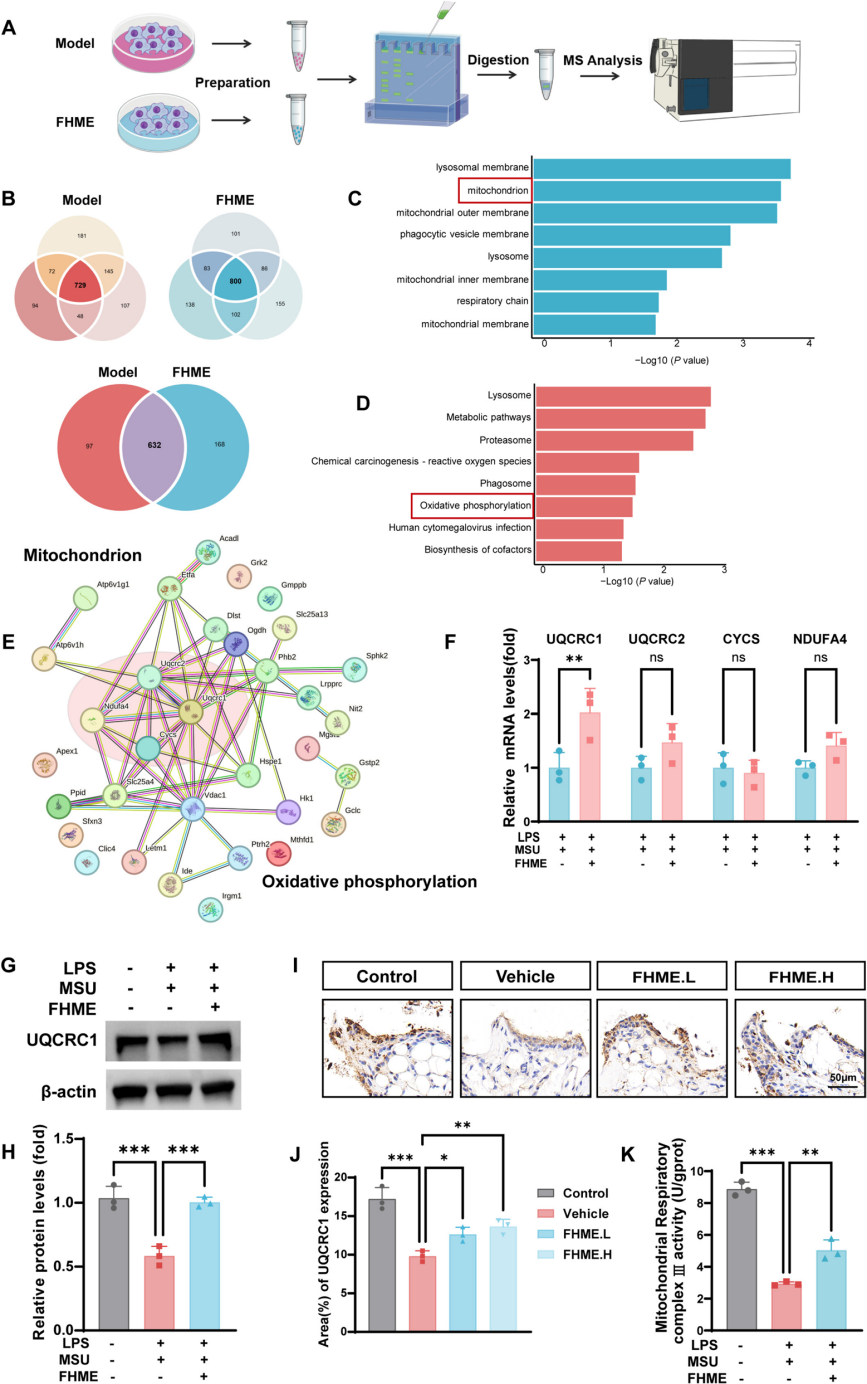
FHME promotes UQCRC1 expression to sustain mitochondrial function. **A** Schematic workflow of the chemical proteomic platform to reveal proteomic changes for FHME treatment in BMDMs. **B** Venn diagram analysis illustrated the number of differential proteins in each set of experiments, overlapped proteins from triplicated samples were defined as the final list in Model or FHME group. **C** The Gene Ontology (GO) terms associated with the cellular component were utilized to conduct functional enrichment clustering analysis on the differentially expressed proteins. **D** A Kyoto Encyclopedia of Genes and Genomes (KEGG) pathway analysis was conducted on the differential proteins. **E** The protein–protein interaction (PPI) analysis of mitochondrion pathway-related proteins and oxidative phosphorylation-associated proteins was visualized by Cytoscape. The red region was used to emphasize the higher degree value. **F** BMDMs were incubated with LPS/MSU with or without FHME for 24 h. Gene levels of UQCRC1, UQCRC2, CYCS and NDUFA4 were determined by qRT-PCR. (G, H) The protein levels of UQCRC1 were detected through western blot (**G**). The quantified protein expression of UQCRC1 in G was normalized to β-actin (**H**). **I** Representative images depicting the immunohistochemical staining of UQCRC1 in the synovial tissues of mice hind paws. (**J**) Statistics and analysis of the percentage of UQCRC1 positive areas in I. **K** Cellular mitochondrial complex III activities were assayed by Coenzyme Q-Cytochrome C Reductase Activity Assay Kit after incubating with LPS/MSU with or without FHME for 24 h. Data were calculated by Student’s t-test, *P < 0.05, **P < 0.01, and ***P < 0.001, ns, no significant differences

To further investigate the mechanisms involved in mitochondrial homeostasis regulation function of FHME, we analyzed the changes of protein level from two groups through LC–MS/MS analysis (Fig. 6A). The Venn diagram illustrated that the Model and FHME groups possess 729 and 800 overlapping proteins respectively. We utilized comparative analysis to reveal 168 differentially expressed proteins in the FHME group (Fig. 6B). GO and KEGG analysis highlighted the biological processes or pathways in which FHME could affect macrophages. Consistent with our data (Fig. 4), the results showed that differential proteins were mainly involved in the regulation of mitochondrion, mitochondrial outer membrane, mitochondrial inner membrane, respiratory chain and the oxidative phosphorylation pathway (Fig. 6C, D). We employed PPI network to analyze the identified proteins in the mitochondrion process and oxidative phosphorylation pathway, which revealed UQCRC1, UQCRC2, CYCS, and NDUFA4 serving as the hubs within two distinct clusters (Fig. 6E). We next used qRT-PCR to examine the impact of FHME on the transcription levels of four protein-encoding genes. Our findings demonstrated that FHME elevated the mRNA levels of UQCRC1, whereas it did not induce any statistically significant changes in the gene expression levels of UQCRC2, CYCS and NDUFA4 (Fig. 6F). The results of western blot showed FHME promotes the expression of UQCRC1 protein (Fig. 6G, H). To further confirm UQCRC1 is the target protein of FHME in vitro, we also analyzed the protein level of UQCRC1 in FHME treated AGA model mice by Immunohistochemical staining. The presentative images and statistical analysis showed that UQCRC1 expression has a significant elevation in the hind paw tissues of AGA mice after FHME treatment (Fig. 6I, J)*.* Due to UQCRC1 is one of evolutionarily conserved core subunit within the mitochondrial respiratory chain Complex III, we ascertained the impact of FHME on mitochondrial Complex III activity in macrophages and discovered that FHME possesses the capability to protect mitochondrial Complex III activity from LPS/MSU treatment (Fig. 6K).

### UQCRC1 is a key mediator in FHME-induced inhibition of M1 macrophage polarization

**Fig. 7:**
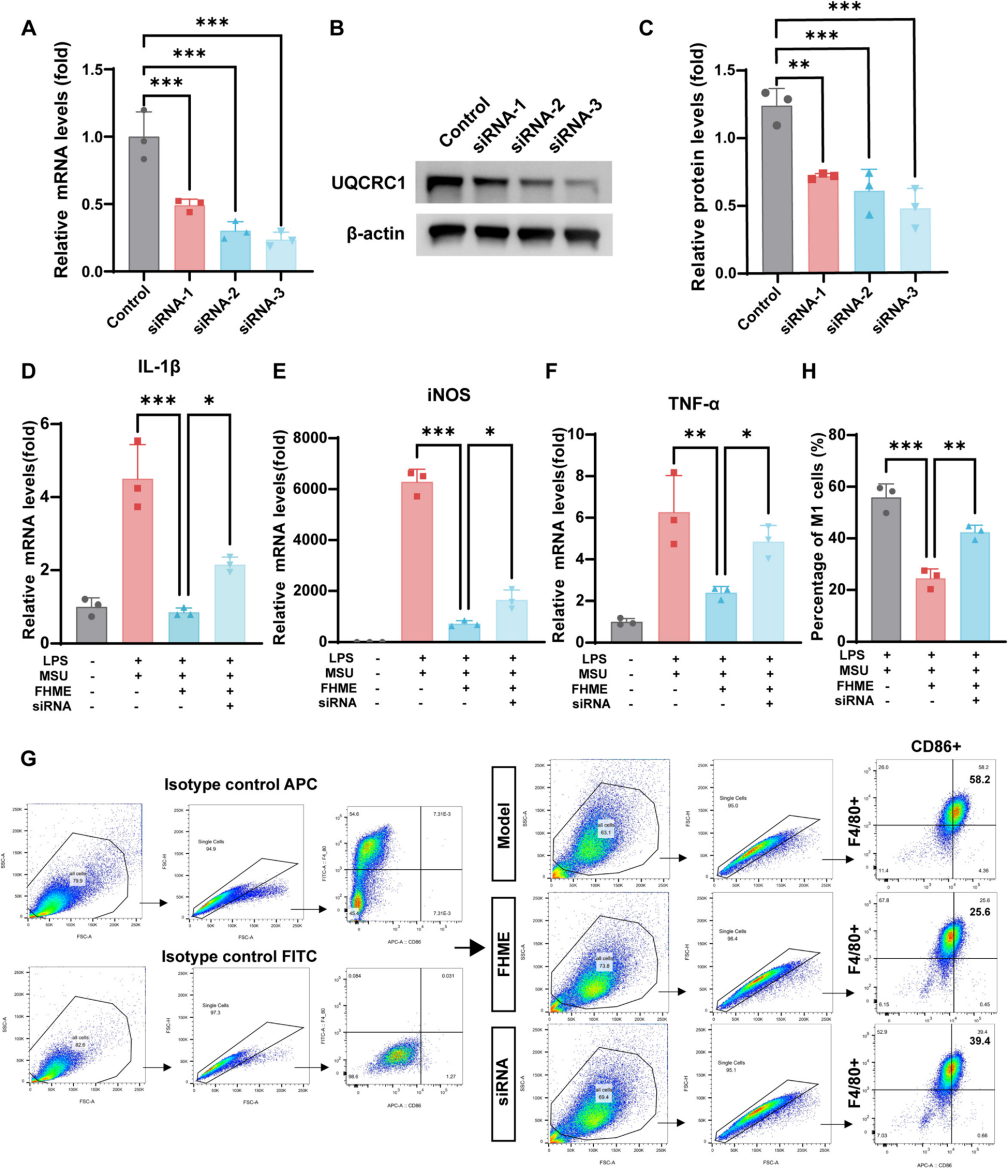
UQCRC1 is a key mediator in FHME-induced inhibition of M1 macrophage polarization. **A**–**C** BMDMs were treated with LPS/MSU in the presence of UQCRC1-siRNA for 24 h. **A** Knockdown efficiency of UQCRC1 siRNA by three different siRNA oligos (UQCRC1-siRNA1, UQCRC1-siRNA2, and UQCRC1-siRNA3) using qRT-PCR. **B** Western blot analysis showed Knockdown efficiency of UQCRC1 siRNA at protein level. (**C**) Gray value analysis of UQCRC1 normalized to β-actin in B. **D**–**H** BMDMs were incubated with LPS/MSU with UQCRC1-siRNA3 for 24 h, followed by an additional 24 h culture with or without FHME. Gene levels of IL-1β (**D**), iNOS (**E**), and TNF-α (**F**) were measured by qRT-PCR analysis. **G**, **H** Isotypes controls for CD86-APC and F4/80-FITC were evaluated. Flow cytometry (**G**) and statistically analysis (**H**) of the proportions of the F4/80^+^CD86^+^ cells relative to the total cell count. Data were calculated by Student’s t-test, *P < 0.05, **P < 0.01, and ***P < 0.001

**Fig. 8:**
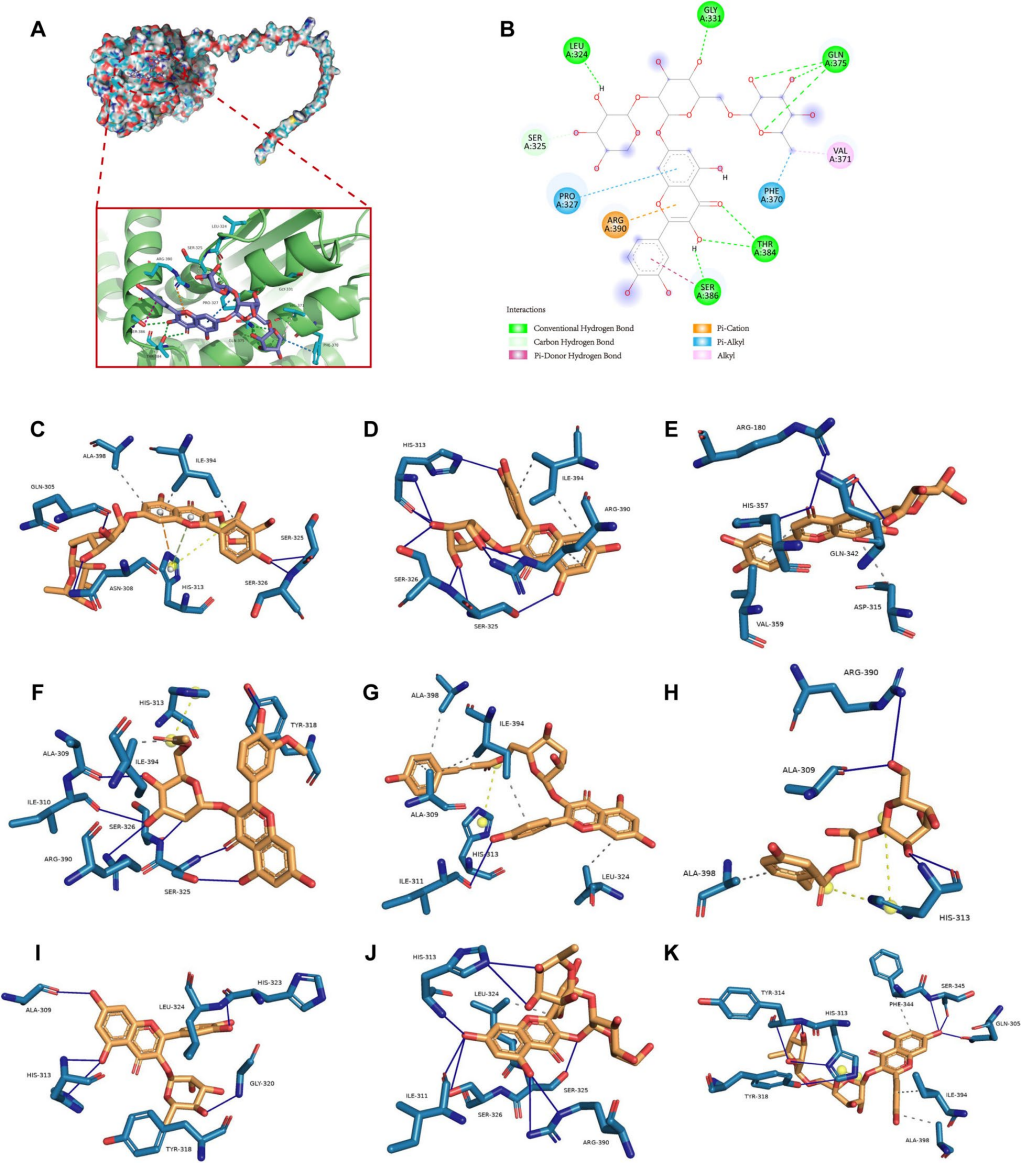
UQCRC1 protein may interact with diverse chemical components of FHME. **A** The predicted 3D interaction between human UQCRC1 protein and myricetin 3-neohesperidoside was shown. Atoms of myricetin 3-neohesperidoside are presented as balls and sticks with carbon atoms in blue, hydrogen atoms in white blue, and oxygen atoms in red. **B** The 2D interaction between myricetin 3-neohesperidoside and UQCRC1. **C**–**K** The interaction diagrams of UQCRC1and Isorhamnetin 3-(6''-acetylgalactoside) (**C**), Hyperin (**D**), Isoorientin (**E**), c cis-p-Coumaric acid 4-[apiosyl-(1–2)-glucoside] (**F**), Tribuloside(**G**), Regaloside D (**H**), Quercitrin (**I**), Kaempferol 3-lathyroside **(J**), Kaempferol 3-lathyroside-7-rhamnoside (**K**) were displayed. The gray dashed line denotes hydrogen bonding, the gray dashed line symbolizes hydrophobic interaction and the yellow dashed line indicates salt bridges

To elucidate the role of UQCRC1 in modulating the inhibitory effect of FEME on M1 macrophage polarization, three different constructs of siRNA were used to knock down UQCRC1 expression. siRNA1/2/3 suppressed mRNA expression of UQCRC1 about 50.84%, 69.69%, 76.32% (Fig. 7A). Besides, the protein expression of UQCRC1 was also decreased, especially when treated with siRNA3 (Fig. 7B, C). We then applied siRNA3 to knockdown UQCRC1 expression in BMDMs and analyzed the anti-inflammatory function of FHME in LPS/MSU-induced M1 macrophage polarization by detecting mRNA levels of IL-1β, iNOS and TNF-α. The results indicated that the anti-inflammatory capability of FHME after UQCRC1 knockdown was diminished (Fig. 8D–F). Flow cytometry also revealed that siRNA3 suspended the reduction of F4/80^+^CD86^+^ cells caused by FHME (Fig. 7G, H). The above results highlighted the protein of UQCRC1 is the key target of FHME to intervene LPS/MSU-induced M1 macrophage polarization.

Finally, to deeply study which compound in FHME targets UQCRC1 protein, we identified 31 chemical compounds in FHME by UHPLC-Q-TOF/MS (S3, TS1). Molecular docking analysis was then performed to investigate the interactions between various components and UQCRC1. The top 10 compounds exhibiting the highest binding affinity were subsequently listed (TS2) and myricetin 3-neohesperidoside showed the highest affinity of − 7.553 kcal/mol. We analyzed the myricetin 3-neohesperidoside-UQCRC1 complex, the following favorable interactions were observed: hydrogen bonds between Leu324, Ser325, Gly331, Gln375, Thr384, Ser386 and myricetin 3-neohesperidoside, electrostatic interaction between Arg390 and myricetin 3-neohesperidoside, and hydrophobic interaction between Pro327 Phe370 Val371 and myricetin 3-neohesperidoside (Fig. 8A, B). Additionally, isorhamnetin 3-(6''-acetylgalactoside), hyperin, isoorientin, cis-p-Coumaric acid 4-[apiosyl-(1–2)-glucoside], tribuloside, regaloside D, quercitrin, kaempferol 3-lathyroside, kaempferol 3-lathyroside-7-rhamnoside were also capable of binding with UQCRC1 (Fig. 8C–K).

## Discussion

*Folium Hibisci Mutabili* has been documented in Chinese Pharmacopoeia (2020 Edition) with efficiencies of relieving herpes zoster, swelling and bruises. Modern pharmacological research demonstrated that FHME possesses antioxidant, antifungal, antiproliferative, anti-inflammatory and neuroprotective properties with low cytotoxicity [21, 27]. For herbal medicine exerting therapeutic effects through the synergistic actions of multiple ingredients targeting various sites [28], we still directly investigated the effects and mechanisms of FHME on AGA in this study. We confirmed the anti-inflammation effect of FHME on MSU-induced AGA model, without any obvious toxic reactions, which was different from colchicine [29]. CIBERSORT algorithm helps us to inspect the underlying mechanism of anti-inflammation effect of FHME. Immunocytes were related to the therapeutic effect of FHME in AGA model.

Macrophages are widely recognized as the primary immune cells involved in AGA and are generally classified into M1 and M2 subtypes depending on their roles in the immune response [30]. M1 macrophages promote inflammation, while M2 macrophages reduce inflammation and aid in tissue repair distinguished by the differential expression of diverse molecules [31]. Our study confirmed an elevated release of IL-1β and an increased proportion of F4/80^+^CD86^+^ macrophages in the ankle joints of AGA mice consistent with previous reports [32]. The analysis of immune infiltration using RNA sequencing results showed that the quantity of M1 macrophages underwent the most remarkable alteration following FHME intervention. The administration of FHME reduced the expression levels of associated inflammatory factors, such as IL-1β, TNF-α and iNOS, and notably decreased the amount of F4/80^+^CD86^+^ cells. Besides, we found that FHME did not affect M2 macrophages’ number and active markers. These findings indicate that FHME exclusively targets one end of the equilibrium between M1 and M2. M1 macrophages may be the more significant immune cells for FHME to treat AGA.

Previous studies have elucidated that M1 macrophages present abnormal mitochondrial morphology and dysfunction [24, 33]. In our research, we found LPS/MSU cultivation of BMDMs caused bubble changes, broken ridges and membrane ruptures in mitochondria, as well as inhibited the aerobic respiration dominated by mitochondria, resulting in cellular energy metabolism relying on anaerobic respiration (Fig. 4). This aligns with prior research demonstrating that M0 macrophages packed with dense, well-developed cristae, whereas M1 macrophages possess fragmented, underdeveloped cristae in mitochondria, and M1 macrophages diminish oxidative phosphorylation (OXPHOS) activity, thereby reigning up increased glycolysis to provide ATP [25, 34]. Considering FHME functions in protecting intracellular mitochondria while inhibiting M1 polarization, we speculated that the maintenance of normal mitochondrial morphology and function may be the one mechanism for FHME to depress M1 macrophage polarization. We used rotenone to shut down mitochondrial respiration and examined the effect of FHME on macrophage polarization. The results showed that the inhibition of M1 macrophage polarization by FHME was mitochondrial-dependent. In short, the increase of mitochondrial OXPHOS activity helping to counteract M1 macrophage inflammatory response was supported by our experiments. FHME depends on mitochondrial homeostasis to weaken the impact of M1 macrophage in AGA.

OXPHOS activity is processed by the electron transport chain, which is composed of transmembrane complexes I to V and cytochrome c in the inner mitochondrial membrane. In the biological process, electrons are passed through a sequence of protein complexes, increasing their reduction potential and releasing energy [35]. To investigate the further mechanism of FHME in alleviating OXPHOS activity, we applied proteomics analysis to explore the hub proteins involved. The results indicated NDUFA4, UQCRC1, UQCRC2 and CYCS were the center of protein interaction. Among of which, NDUFA4 is an important subunit in Complex IV, capable of transferring electrons from NADH to the respiratory chain [36]. UQCRC1 and UQCRC2 are key subunits of mitochondrial respiratory complex III and play critical roles in transferring electrons from coenzyme Q sequentially to cytochrome C bound to the outer surface of the mitochondrial inner membrane [37, 38]. CYCS encodes cytochrome C, which transfers electrons from coenzyme Q to oxygen. It is also one of the key substances that initiate cell apoptosis [39]. However, the roles of these four factors in the pathogenesis of AGA have not been reported. To concentrate on the elements most significantly influenced by FHME, qRT-PCR was employed to measure mRNA alterations in four factors post-FHME treatment. FHME only increased the gene expression of UQCRC1. Subsequently, we confirmed that the activity of mitochondrial respiratory complex III was significantly increased with the involvement of FHME. UQCRC1 deregulation has been reported to be involved in a variety of disorders, such as Parkinson's disease and pancreatic cancer [23, 39]. Up to date, the anti-inflammatory properties of UQCRC1 have not been reported. Against this background, we silenced the expression of UQCRC1 by siRNA and found that the knockdown of UQCRC1 in macrophages increased the release of inflammatory cytokines and promoted an increase in the number of M1 macrophages. FHME rescued the inhibition of M0 to M1 polarization. Therefore, we highlighted that FHME alleviates mitochondrial function, as well as inhibits macrophage polarization towards M1 closely relating to UQCRC1 expression. Thus, UQCRC1 may be an all-new target for treating AGA.

To further investigate the interaction between UQCRC1 and FHME components, we explored the binding of FHME's components to UQCRC1 by molecular docking. We first identified 31 compounds from FHME, which is consistent with the results demonstrated in previous studies that flavonoids, organic acids and coumarins are the main components of FHME [40]. The molecular docking results showed that myricetin 3-neohesperidoside, isorhamnetin 3-(6''-acetylgalactoside), hyperi, isoorientin and so on, had higher binding affinity with UQCRC1 protein. These findings suggest that these compounds may play a crucial role in the therapeutic effects of FHME by modulating UQCRC1 activity, impacting mitochondrial function, and subsequently influencing inflammatory responses. Further experimental validation is needed to confirm these interactions and to elucidate the precise mechanisms through which FHME's components exert their immunomodulatory effects.

## Conclusion

**Fig. 9:**
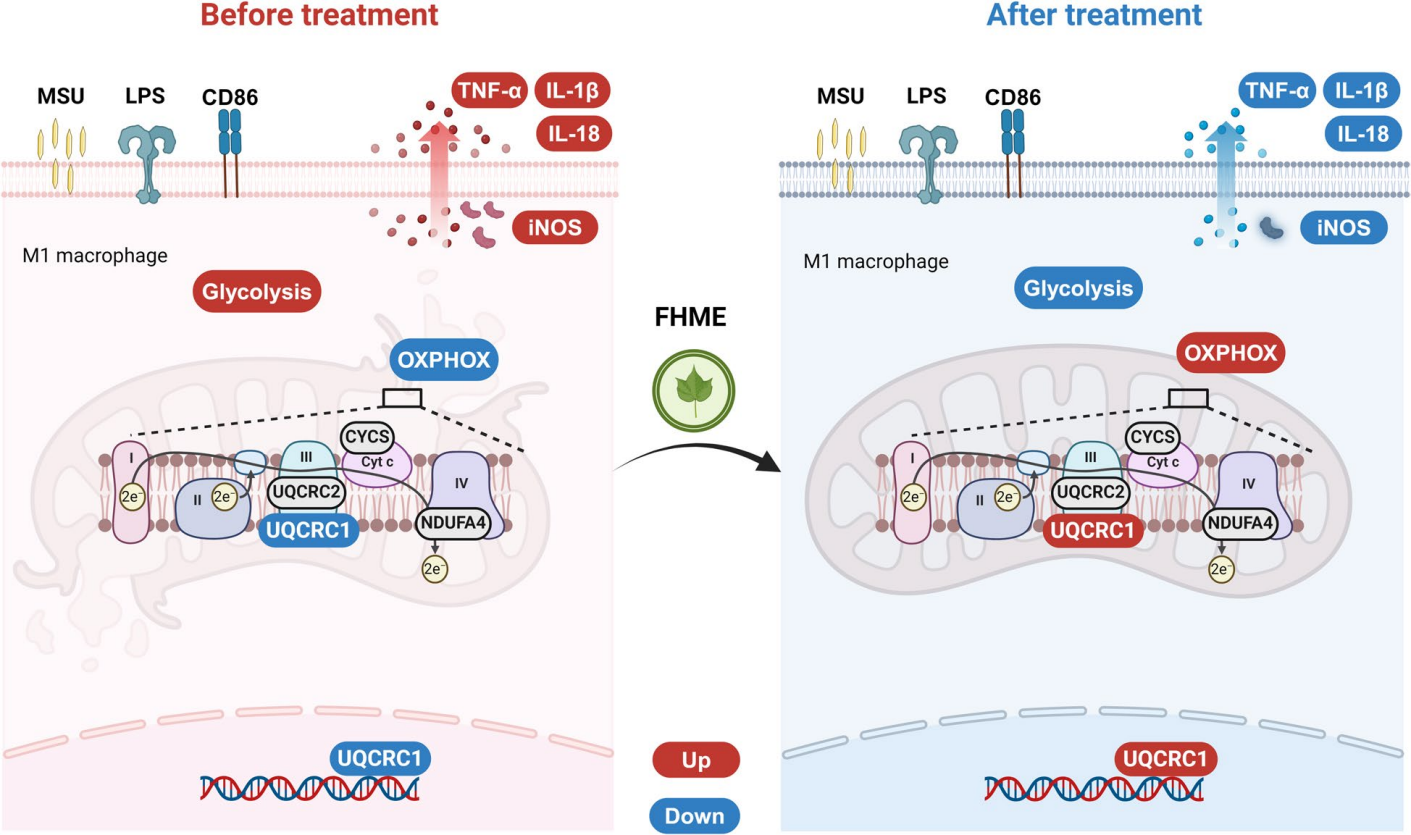
The diagram illustrates the mechanisms underlying FHME suppresses macrophage inflammatory response through restoring mitochondrial function

This is the first study to investigate the effects and mechanisms of FHME on AGA. FHME maintains mitochondrial morphology and function to inhibit macrophage polarization towards M1, which is closely related to UQCRC1 expression. Our findings provide insights into the therapeutic potential of *Folium Hibisci Mutabili* as a safe and effective approach in AGA. In addition, this study shows that UQCRC1 may be a novel target for the treatment of AGA (Fig. 9).

## Supplementary Information

Supplementary material 1

Supplementary material 2

Supplementary material 3

Supplementary material 4

Supplementary material 5

## Data Availability

The datasets used and analysed during the current study are available from the corresponding author on reasonable request.

## Abbreviations

AGA: Acute gout arthritis
ALT: Alanine aminotransferase
AST: Aspartate aminotransferase
BMDMs: Bone marrow-derived monocytes
BUN: Blood urea nitrogen
CREA: Creatinine
ECAR: Extracellular acidification rate
ELISA: Enzyme-linked immunosorbent assay
FHME: *Folium Hibisci Mutabilis* Extract
HE: Hematoxylin–eosin
IF: Immunofluorescence
IHC: Immunohistochemical
IL-18: Interleukin 18
IL-1β: Interleukin 1β
iNOS: Inducible nitric oxide synthase
LPS: Lipopolysaccharides
M-CSF: Macrophage Colony-Stimulating Factor
MSU: Monosodium urate
NO: Nitrogen monoxide
OCR: Oxygen consumption rate
OXPHOS: Oxidative phosphorylation
PCA: Principal component analysis
PWT: Paw withdrawal thresholds
qRT-PCR: Quantitative real-time PCR
TEM: Transmission electron microscope
TNF-α: Tumor necrosis factor-α
UQCRC1: Ubiquinol Cytochrome c Reductase Core Protein I
