# Supplementary figures and images for: Folium Hibisci Mutabilis extract suppresses M1 macrophage polarization through mitochondrial function enhancement in murine acute gouty arthritis
Source: Chin Med. 2025 Feb 28;20:28. doi: 10.1186/s13020-025-01081-6 (PMC11869456; doi:10.1186/s13020-025-01081-6)

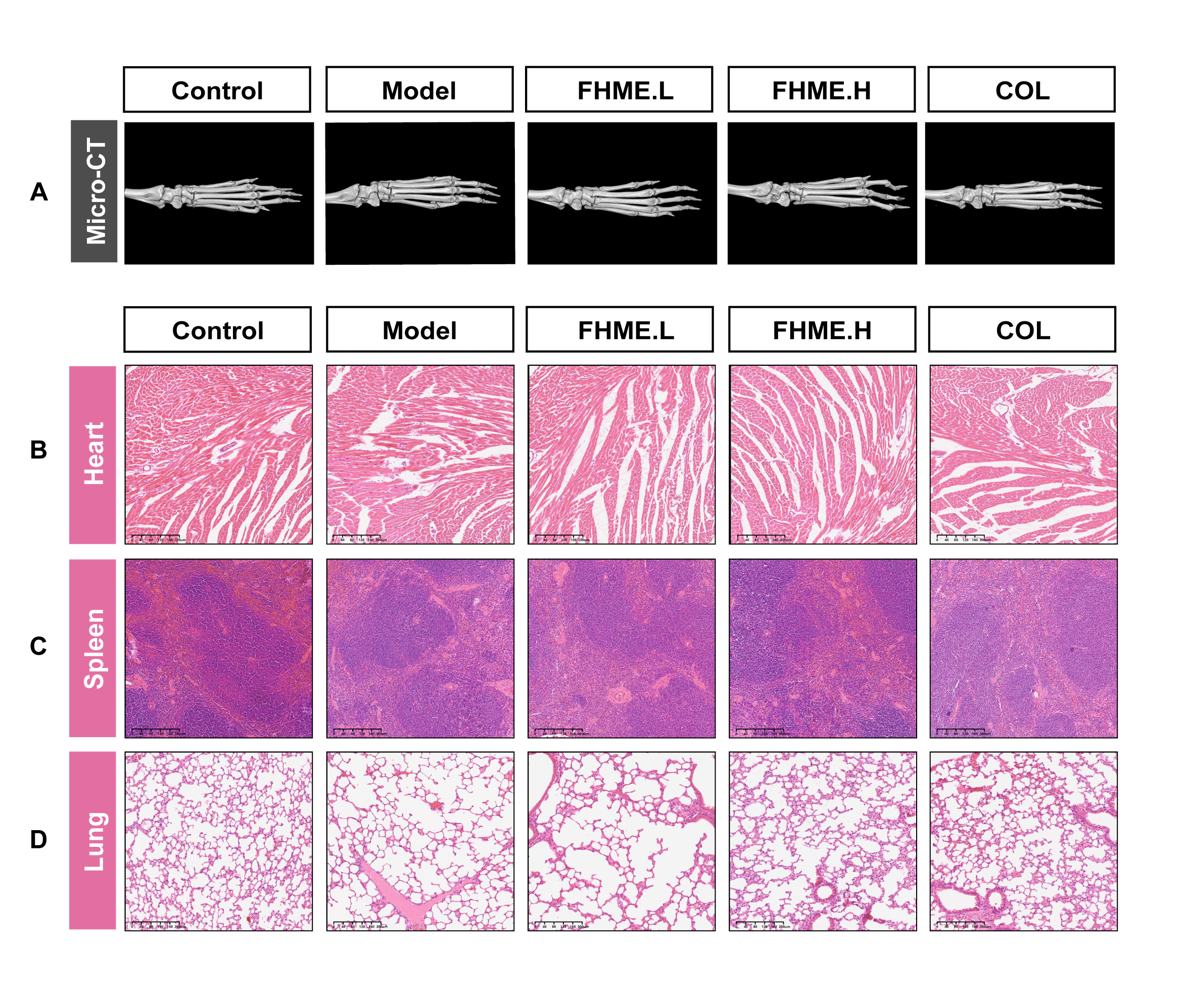

Supplement: Supplementary file 1 — Supplementary material 1 [file 13020_2025_1081_MOESM1_ESM.tif]

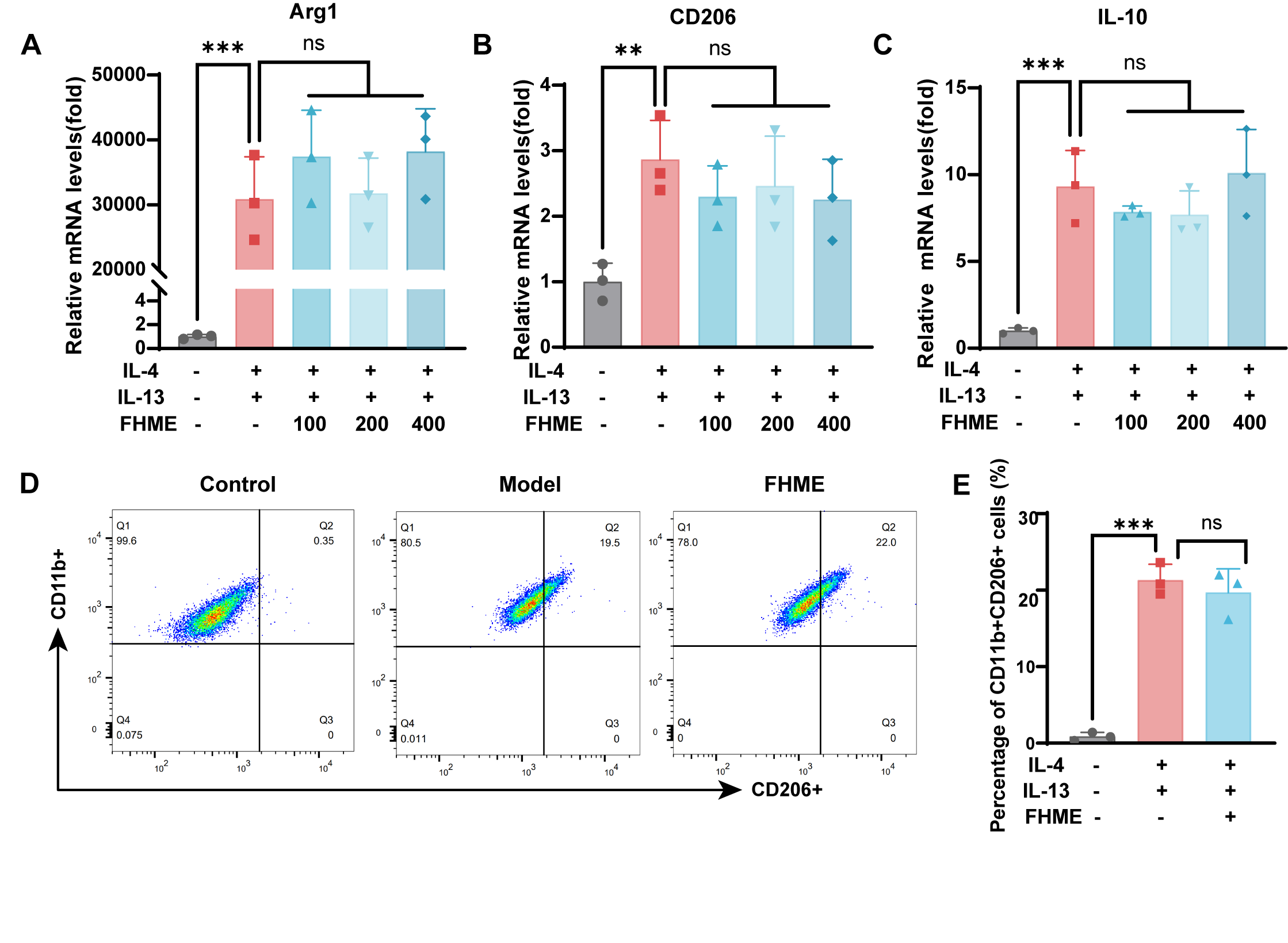

Supplement: Supplementary file 2 — Supplementary material 2 [file 13020_2025_1081_MOESM2_ESM.tif]

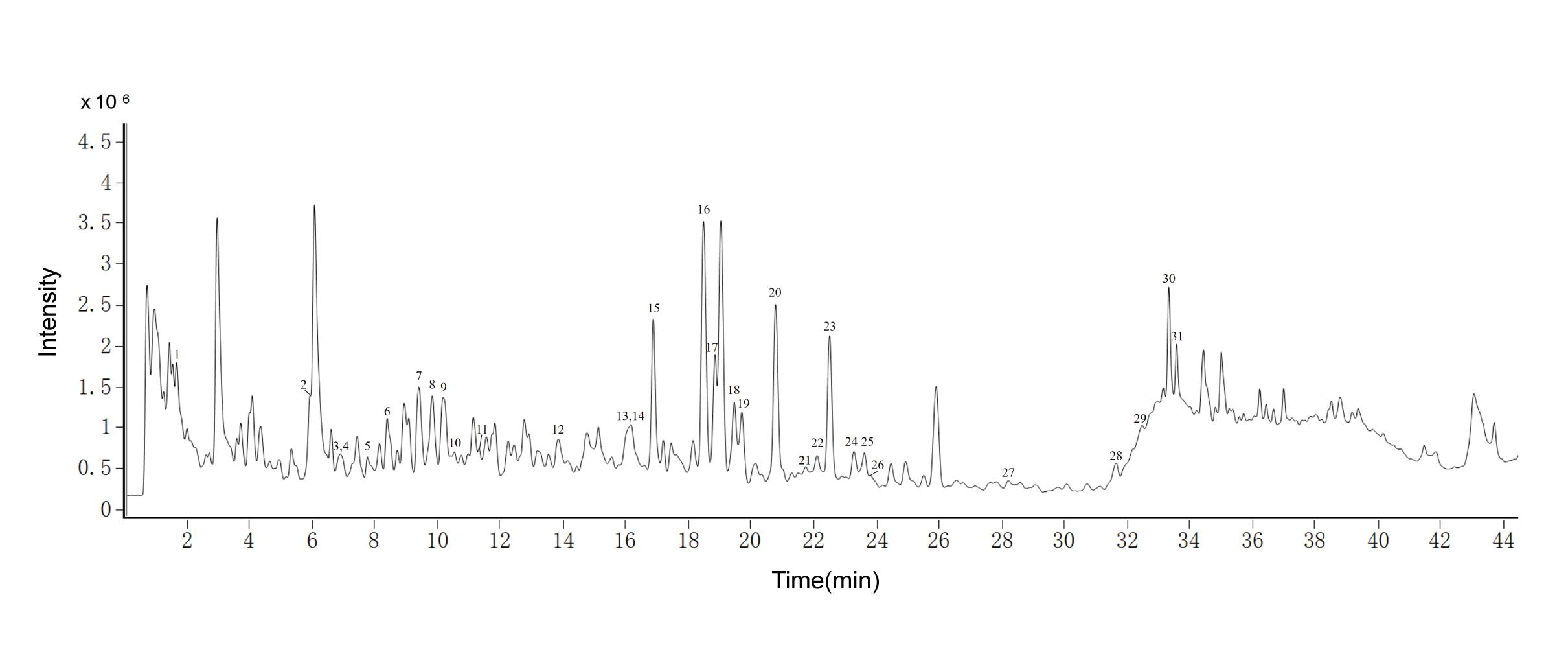

Supplement: Supplementary file 3 — Supplementary material 3 [file 13020_2025_1081_MOESM3_ESM.tif]
