# Supplementary material for: Folium Hibisci Mutabilis extract suppresses M1 macrophage polarization through mitochondrial function enhancement in murine acute gouty arthritis
Source: Chin Med. 2025 Feb 28;20:28. doi: 10.1186/s13020-025-01081-6 (PMC11869456; doi:10.1186/s13020-025-01081-6)
Supplement: Supplementary file 4 — Supplementary material 4 [file 13020_2025_1081_MOESM4_ESM.docx]

**Supplementary Figure Captain**

**Figure S1.** Representative radiographs from the hind paws of each group under micro-CT (A). Representative H&E-stained images of the heart (B), spleen (C), and lung (D) in the Control group, Vehicle group, FHME.L group, FHME.H group, and COL group.

**Figure S2.** M-CSF and LPS were incubated with BMDMs in the presence of increasing doses of FHME (100, 200 and 400µg/mL) for 24h, and gene levels of (A) Arg1, (B) CD206 and (C) IL-10 were measured by qRT-PCR. (D) BMDMs were cultured with M-CSF and LPS in the presence of 200µg/mL FHME for 24h, CD11b and CD206 were labeled by antibodies and detected by flow cytometry. (F) Statistics analysis of the percentage of CD11b ^+^ CD11b ^+^ cells in total cells.

**Figure S3.** Total ion chromatograms of *Folium Hibisci Mutabilis* extract (FHME) in negative ion mode. UHPLC/Q-TOF-MS was used to illustrate the major components of FHME extract and 31 compounds were identified.
