# Supplementary material for: Folium Hibisci Mutabilis extract suppresses M1 macrophage polarization through mitochondrial function enhancement in murine acute gouty arthritis
Source: Chin Med. 2025 Feb 28;20:28. doi: 10.1186/s13020-025-01081-6 (PMC11869456; doi:10.1186/s13020-025-01081-6)
Supplement: Supplementary file 5 — Supplementary material 5 [file 13020_2025_1081_MOESM5_ESM.docx]

**Table S1.** List of identified components in FHME through UHPLC/Q-TOF-MS analysis

| Number | Retention time (min) | Name | Molecular formula | [M+H]+/[M-H]- | Adduct |
| --- | --- | --- | --- | --- | --- |
| 1 | 1.675 | Fumaric acid | C4H4O4 | 115.0035 | [M-H]- |
| 2 | 5.921 | Esculin | C15H16O9 | 385.0767 | [M+FA-H]- |
| 3 | 6.809 | Uralenneoside | C12H14O8 | 285.0608 | [M-H]- |
| 4 | 6.843 | Salicylic acid | C7H6O3 | 137.0243 | [M-H]- |
| 5 | 7.765 | Salicylic acid-beta-D-glucoside | C13H16O8 | 299.0763 | [M-H]- |
| 6 | 8.414 | Ermanine | C17H14O6 | 359.0748 | [M+FA-H]- |
| 7 | 9.427 | p-Hydroxycinnamic acid | C9H8O3 | 163.0398 | [M-H]- |
| 8 | 9.735 | Esculetin | C9H6O4 | 177.0188 | [M-H]- |
| 9 | 10.156 | Caffeic acid | C9H8O4 | 179.0345 | [M-H]- |
| 10 | 10.429 | Fraxin | C16H18O10 | 415.0877 | [M+FA-H]- |
| 11 | 11.203 | cis-p-Coumaric acid 4-[apiosyl-(1-2)-glucoside] | C20H26O12 | 457.134 | [M-H]- |
| 12 | 13.867 | Phenylacetaldehyde | C8H8O | 119.0497 | [M-H]- |
| 13 | 16.076 | Kaempferol 3-lathyroside | C26H28O15 | 625.1391 | [M+FA-H]- |
| 14 | 16.076 | Myricetin-3-neohesperidoside | C27H30O17 | 625.1391 | [M-H]- |
| 15 | 16.873 | Quercetin 3-(2G-xylosylrutinoside) | C32H38O20 | 741.1858 | [M-H]- |
| 16 | 18.478 | Rutin | C27H30O16 | 609.1434 | [M-H]- |
| 17 | 18.854 | Kaempferol 3-lathyroside-7-rhamnoside | C32H38O19 | 725.1919 | [M-H]- |
| 18 | 19.355 | 4-Hydroxy-3-methoxystyrene | C9H10O2 | 195.0656 | [M+FA-H]- |
| 19 | 19.719 | Hyperin | C21H20O12 | 463.086 | [M-H]- |
| 20 | 20.778 | Biorobin | C27H30O15 | 593.1484 | [M-H]- |
| 21 | 21.760 | Isoorientin | C21H20O11 | 447.0917 | [M-H]- |
| 22 | 22.121 | Azelaic acid | C9H16O4 | 187.0967 | [M-H]- |
| 23 | 22.508 | Astragalin 7-rhamnoside | C27H30O15 | 593.1485 | [M-H]- |
| 24 | 23.282 | Quercitrin | C21H20O11 | 447.0913 | [M-H]- |
| 25 | 23.487 | Regaloside D | C18H24O10 | 459.153 | [M+Hac-H]- |
| 26 | 23.612 | Tamarixetin 3-rutinoside | C28H32O16 | 623.1584 | [M-H]- |
| 27 | 28.166 | Isorhamnetin 3-(6''-acetylgalactoside) | C24H24O13 | 519.1135 | [M-H]- |
| 28 | 31.430 | Sebacic acid | C10H18O4 | 201.1127 | [M-H]- |
| 29 | 32.504 | Quercetin | C15H10O7 | 301.0345 | [M-H]- |
| 30 | 33.323 | Tiliroside | C30H26O13 | 593.1283 | [M-H]- |
| 31 | 33.574 | Tribuloside | C30H26O13 | 593.1285 | [M-H]- |
